# Failure of Intravenous Silibinin Monotherapy to Prevent Hepatitis C Genotype 2A Liver Graft Reinfection

**DOI:** 10.5812/hepatmon.6135

**Published:** 2012-06-30

**Authors:** Alessio Aghemo, Sherrie Bhoori, Stella De nicola, Vincenzo Mazzaferro, Massimo Colombo

**Affiliations:** 1 Angela Maria e Antonio Migliavacca Center for Liver Disease, First Division of Gastroenterology, Fondazione IRCCS Ca’ Granda ospedale Maggiore Policlinico, University of Milan, Milan, Italy; 2 Hepato-oncology Group, Department of Gastroenterology and Surgery, IRCCS national Cancer Institute, Milan, Italy

**Keywords:** Hepacivirus, Silybin, Liver Transplantation

## Abstract

**Background:**

Hepatitis C virus (HCV) recurrence after orthotopic liver transplantation (OLT) remains a serious problem in the clinical management of post-oLT patients. Recently, two case reports have described successful prevention of HCV liver graft reinfection with intravenous silibinin (SIL) monotherapy in two carriers of genotype 3a and 1a/4 HCV. Based on these findings, we decided to offer such a therapy to a 65 year old woman on the oLT list.

**Case Presentation:**

A 65 year old patient with HCV 2a cirrhosis, a previous relapse to PegIFn and Rbv therapy, was listed for oLT due to hepatocellular carcinoma. She started SIL monotherapy 24 hours before oLT. After an initial HCV-RnA decline following surgery,a progressive HCV RnA increase was observed. For this reason, SIL was stopped after 15 days of monotherapy.

**Conclusions:**

SIL has multiple anti-HCV mechanisms of action, most of them have been characterized in vitro only. Our case report shows that the antiviral effect of SIL might be HCV genotype dependent, as recently suggested by a study, showing no effect of SIL on the HCV-2a subgenomic replicon model. our case reinforces the need for controlled studies to assess the efficacy of silibinin therapy in HCV infected patients before it can be broadly used in all clinical settings.

## 1. Background

Hepatitis C virus (HCV) recurrence after orthotopic liver transplantation (OLT) remains a serious problem in the clinical management of post-OLT patients as it is associated with substantial morbidity, mortality and graft loss [1][2]. While in the anhepatic phase of OLT, hepatitis C viremia can become undetectable, HCV replication may reoccur within hours or days following OLT in patients who are HCV-RNA positive at surgery. Unfortunately, treatment of chronic HCV in liver transplant recipients is suboptimal, as no prophylactic therapy is available and combination therapy with Pegylated interferon (PegIFN) and ribavirin (Rbv) which is the current gold standard for chronic HCV infection, is associated with an increased incidence of infections and rejections [3]. Moreover, the treatment is poorly tolerated, requires frequent Peg-IFN and/or ribavirin dose reductions to manage its related cytopenias. For these reasons, there is much clinical interest in developing alternatives therapies. The first generation of Directly Acting Antivirals (DAA) unfortunately will not change this scenario that much, as they require association with PegIFN and Rbv to be effective and may actually be harmful in post-OLT patients due to the drug-drug interaction with the concurrent immunosuppressive regimen [4][5][6]. Concerning this as a background, much enthusiasm was induced following the reports by Neumann et al. and Beinhardt et al. of successful prevention of HCV liver graft reinfection with an intravenous silibinin monotherapy in two carriers of genotype 3a and 1a/4 HCV [7][8]. The rationale to use silibinin monotherapy in OLT patients was first demonstrated by Ferenci et al. in a pilot study in previous non-responders to a course of Peg-IFN/RBV, based on the anti-HCV effect of a 15-20 day course of intravenous silibinin administration [9].

Based on these findings, we decided to offer such a therapy to a 65 year old woman on the OLT list at the Liver Center of National Cancer Institute.

## 2. Case Presentation

**Figure 1:**
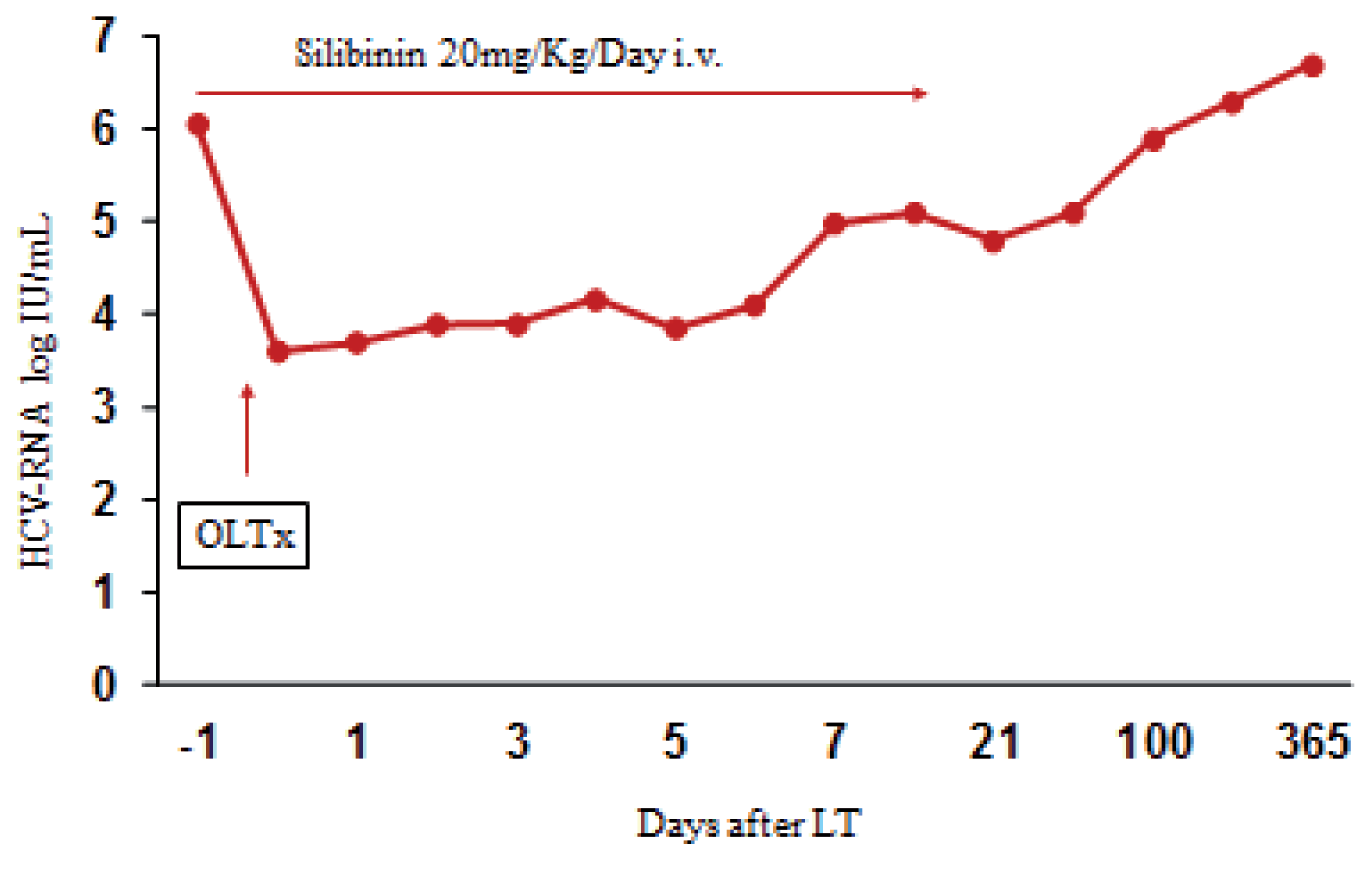
HCV RNA Kinetics After Liver Transplantation (LT) During 14 Days of Silibinin IV Monotherapy and Subsequent Follow-up (HCV RNA Levels Measured by Abbott Real Time HCV Assay)

The patient was diagnosed as having HCV-related cirrhosis, genotype 2a, in January 2003 and from that point, was followed up regularly in another hospital with liver function tests and abdominal ultra sounds every six months without being offered antiviral treatment. In April 2009, following the detection of a 25 mm hepatocellular carcinoma (HCC) in segment 4, the patient was referred to our center and was successfully treated with radio frequency ablation (RFA). She was then offered Pegylated Interferon-alfa 2a 180 mcg/week plus ribavirin 800 mg/day. Serum HCV RNA became undetectable with a RT-PCR assay (lower limit of detection of 12 IU/mL) for the first time at week eight, and remained undetectable until the end of therapy (week 24). During the post-treatment follow-up, the patient had a virological and biochemical relapse, while the CT scan showed a single HCC early recurrence (Ø 12 mm) that was ablated by RFA. At this point the patient was listed for OLT. Silibinin (Legalon SIL®, Rottapharm-Madaus) at the dose of 20mg/Kg body weight was started intravenously 24 hours before OLT with serum HCV RNA levels being 1,932,431 IU/mL. At the time of OLT, the HCV RNA viral load was 1,673,380 IU/mL which eight hours following OLT declined to 4,458 IU/mL. Since serum HCV RNA values progressively increased during the daily infusions to reach 904,464 IU/mL at the 15th day of silibinin monotherapy (Figure 1), we decided to stop anti-HCV treatment. In the following six months post-OLT, no recurrence of HCC was observed, while the HCV-RNA load progressively increased to reach 6 log IU/ml values. In order to investigate ALT and AST increase, we performed a liver biopsy at four months post-OLT demonstrating HCV recurrence, no cirrhosis and no signs of rejection.

## 3. Discussion

Silymarin is an extract from the seeds of the milk thistle plant Silybum marianum, and is one of the most popular herbal drugs used in chronic liver disease due to a supposed anti-oxidant effect. Data derived from the HALT-C trial showed that at the baseline, 34% (348/1049) of patients reported silymarin oral assumption in their medical history, with 16% (170/1049) of them currently taking the drug [10]. In the same trial, the final analysis reported no clinical impact of oral silymarin on HCV infection in terms of fibrosis liver progression, liver disease-related death, hepatic encephalopathy, hepatocellular carcinoma, spontaneous bacterial peritonitis or variceal haemorrhage [11]. One of the potential reasons behind this apparent lack of effect of silymarin is that the oral formulation has low bioavailability due to its insolubility in aqueous solution.

Silibinin is the largest component extract from silymarin, consisting of the flavolignans silybin A and B. A soluble version of silibinin, SIL that has been developed by chemical modification in vitro is currently used intravenously for the treatment of hepatic intoxication by Amanita phalloides mushrooms. Some studies demonstrated a potent effect of SIL in reducing HCV RNA serum load in vitro [12]. Hypothesizing an antioxidant effect of SIL, Ferenci et al. tested this product in patients with chronic hepatitis C and a previous non response to Peg-IFN and ribavirin. In the first study, SIL was used at the fixed dose of 10 mg/kg/day in 16 patients for seven days. In a subsequent dose-finding study, 20 patients received either 5, 10, 15, or 20 mg/kg/day of SIL. In both studies Peg-IFN/RBV therapy was started on the eighth day of SIL therapy. Daily HCV RNA measurement showed a SIL dose dependent viral load decrease with HCV-RNA undetectability being reached in 7/20 patients with SIL monotherapy. Following this breakthrough, Neumann and Beinhardt employed SIL as a preventive therapy in HCV patients undergoing liver transplantation. Neumann treated a 57 year old patient, infected with HCV-3a that was listed for OLT for cirrhosis with hepatocellular carcinoma. The patient previously failed Peg-IFN and Rbv therapy. SIL was started at dose of 1400 mg/day immediately after OLT and was infused for 14 days. Serum HCV-RNA became undetectable in a few days and the patient was HCV recurrence free at 160 days following the start of therapy. Differently, in the second report, an HCV 1a/4 patient received SIL for 15 days at the dose of 20 mg/Kg/day before OLT and until 25 days after OLT. Serum HCV RNA negativity was reached at day 10, with HCV-RNA negativity being maintained until 168 days post-OLT follow-up. The anti-HCV mechanisms of action of SIL are many. Indeed, while SIL shows no direct liver cito-toxicity, it may potentially alter the HCV lifecycle trough interaction with multiple targets. Firstly, Morishima et al. demonstrated the anti-inflammatory and antiviral effects of this product, by reporting inhibited expression of tumor necrosis factor alpha in anti-CD3 stimulated human peripheral blood mononuclear cells and of nuclear factor kappa B-dependent transcription [13]. Subsequently, it was found that silymarin and SIL inhibit HCV NS5B RNA-dependent RNA polymerase activity [14], a key enzyme in the HCV life cycle that is the target of second generation DAAs. Recently, Wagoner et al. described an effect on the fusion of HCV pseudoparticles (HCVpp) in a dose-dependent fashion with SIL, preventing HCV cell entry and cell to cell spread of the virus. Using the JFH1 strain infection model cell culture system, they also showed that SIL inhibited 1b replicons of JFH1 infection but not 2a subgenomic replicons [15]. This in vitro demonstration can, partially, explain why silibinin monotherapy failed to prevent liver graft reinfection in our HCV-2 patient. Moreover if SIL is an NS5B polymerase inhibitor, it is important to bear in mind that this class of drugs can be divided into two classes: nucleoside analogues (NUCs) and non-nucleoside analogues (NNUCs) [16]. The first class by definition is pan-genotypic as it blocks the polymerase activity by incorporation of the analogue in place of a nucleotide, hence stopping the polymerization of the nascent RNA. The second class acts through NS5B binding that determines allosteric changes that preclude polymerase activity. The potential binding sites are many and potentially different among HCV genotypes, for this reason, these drugs do not show broad anti-HCV activity. Indeed, the inhibition of the specific RNA-dependent RNA polymerase activity is unequally distributed across the HCV genotypes being ranked in the following descending order: 1b, 6a > 2a, 3a, 4a, 5a > 1a [17]. To show whether SIL acts trough the NNUCs pathway needs demonstration, but actually it explains our data of failure to inhibit HCV RNA replication in an HCV-2 patient.

## 4. Conclusions

In the wait for better characterization of the precise anti-HCV mechanisms of SIL, our conclusion somewhat mitigate the initial clinical enthusiasm for this drug in the OLT setting whilst reinforcing the need for controlled studies on intravenous silibinin therapy in HCV infected patients especially in patients on the OLT waiting-list.
